# Strength Is Not Health

**Published:** 1885-03

**Authors:** Nathan Allen


### STRENGTH IS NOT HEALTH.

"Health and strength are not synonymous terms. A person may have great strength in his limbs or in certain muscles about the body, but really not have good health. It is altogether a mistaken idea to suppose that physical exercises have for the object the attainment of strength. There are other tissues and organs in the human system besides the muscular; and the healthy action of the lungs and stomach is far more important than great strength in the arms, legs, or the back. It is here, in this general exercise of all the muscles and parts of the body, that a well regulated system of gymnastics has its great excellence. It aims to produce just that development of the human system upon which good health is peranently based, described by an English writer as follows: 'Health is the uniform and regular performance of all the functions of the body arising from the harmonious action of all its parts.'—a physical condition implying that all are sound, well-fitting and well-matched. Some minds do not look far enough into life to see this distinction, or to value it if seen; they fix their eyes longingly upon *strength*—upon strength *now*, and seemingly care not for the power to work long, to work well, to work successfully hereafter, which is *health*."—*Dr. Nathan Allen.*

